# Parasite community structure of *Sardina pilchardus* in the Black Sea: diversity, host factors and seasonal dynamics

**DOI:** 10.1017/S0031182026102455

**Published:** 2026-07

**Authors:** Arzu Güven, Türkay Öztürk

**Affiliations:** 1 Department of Aquaculture, Malatya Turgut Özal Üniversitesi, Malatya, Türkiye; 2 Aquaculture and Disease, Sinop Universitesi, Sinop, Türkiye

**Keywords:** Black Sea, community structure, fish parasites, *Sardina pilchardus*, Türkiye

## Abstract

The parasite fauna of *Sardina pilchardus* from the Black Sea coast of Türkiye was investigated. The parasitological survey was conducted on a total of 136 fish collected monthly from October 2024 to May 2025. A total of 7 parasite species were recovered: 5 digeneans (comprising 3 adult stages and 2 metacercarial stages), 1 larval nematode and 1 coccidian. The identified taxa included the adult digeneans *Pronoprymna ventricosa, Lecithaster confusus* and *Aphanurus stossichii*, metacercariae *Stephanostomum* sp. and *Renicola* sp., nematode *Hysterothylacium aduncum*, and coccidian *Eimeria sardinae.* Prevalence (%) and infection intensity indices were determined for each parasite species. Overall infection prevalence (82.35%), mean intensity (8.28 ± 0.74) and mean abundance (6.82 ± 0.67) were calculated. Infection parameters for each parasite species were determined and statistically compared across fish length groups, seasons, sex and host body condition factor. The results revealed that parasite abundance and intensity varied significantly among fish length groups and seasons, whereas no significant correlations were found with host body condition or fish sex. Spearman’s correlation analysis was performed to examine co-occurrence patterns among parasite species. A moderate positive correlation was detected between *H. aduncum* and *Stephanostomum* sp. (*r* = 0.494, *P* < 0.01). In contrast, associations among other parasite species were weak or negligible, indicating largely independent occurrence. Except for *H. aduncum*, all identified species represent new host records for this species in the Black Sea and Türkiye. Notably, *Renicola* sp. constitutes the first record of this genus for the parasite fauna of Türkiye.

## Graphical abstract

**Figure:**
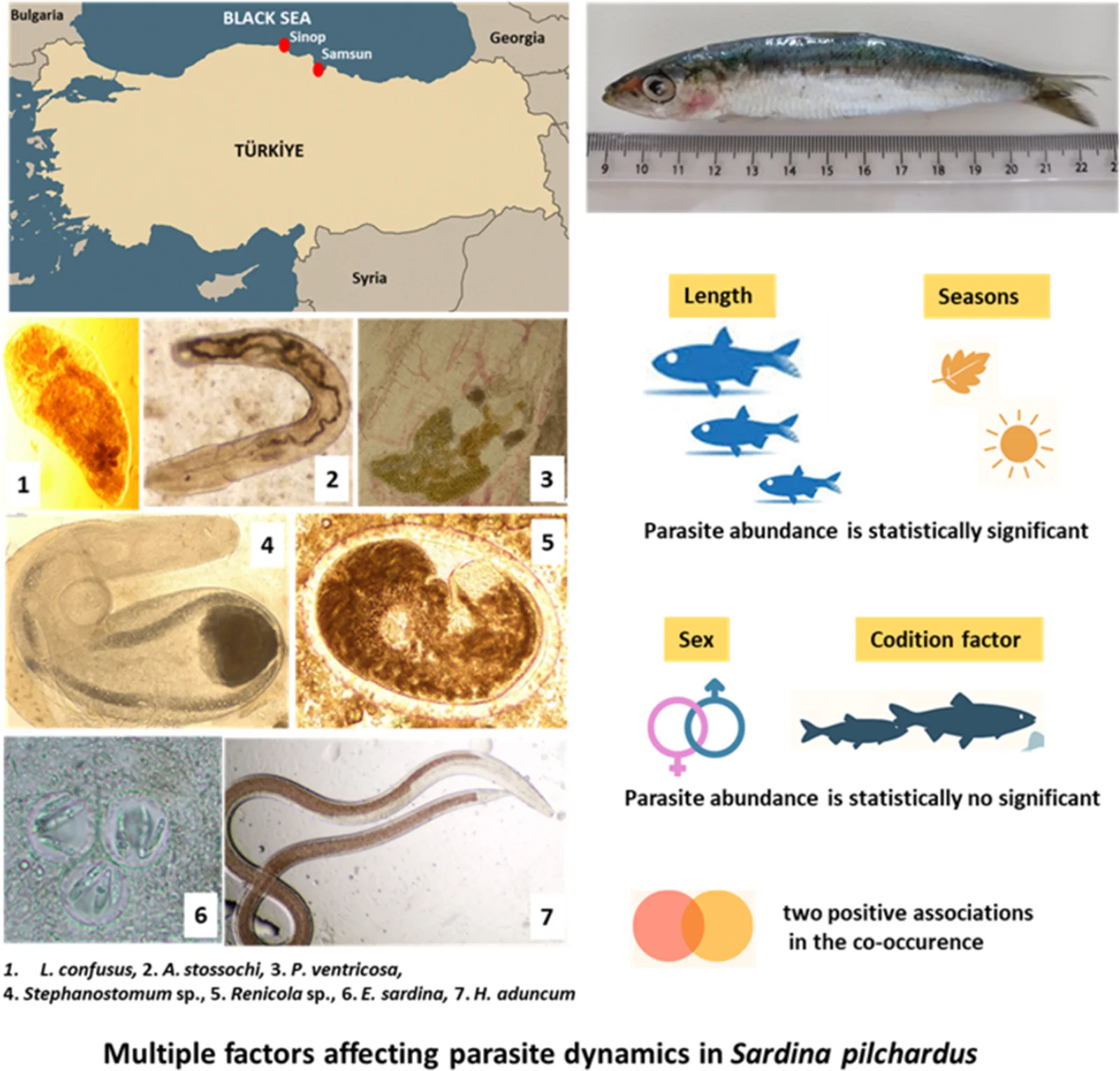

## Introduction

Members of the order Clupeiformes account for a large proportion of pelagic marine fish catches and include small pelagic species such as anchovies and sardines. Their small size and low trophic position further contribute to their ecological and economic importance (Cury et al., 2000; Barros, 2011; Pikitch et al., 2014). The sardine, *Sardina pilchardus* Walbaum, 1792, is consumed as food for humans, particularly in the NorthEast Atlantic and the Mediterranean basin, including the Black Sea (Dalgıç and Ceylan, 2012; Marzoug et al., 2012).

This species is an extremely important seafood source as it is rich in both polyunsaturated fatty acids and essential amino acids (Costalago and Palomera, 2014; Šimat et al., 2020). This species is distributed along all coasts of Türkiye; however, fishing pressure on the populations of this species has increased in recent years (Voulgaridou and Stergiou, 2003; Erdoğan et al., 2010). In addition to basic factors such as fishing pressure and increasing temperature, reduced food availability and parasite infection have been suggested as potential contributors to declines in the abundance of this species, which plays an important role in the food web (Barber et al., 2000; Ramírez et al., 2018; Pennino et al., 2020; Fernández-Corredor et al., 2021; Frigola-Tepe et al., 2022). Parasite infections negatively affect fish health, reproductive efficiency and consequently population dynamics. This is a critical biological determinant potentially affecting stock status in sardines, as in other fish (Barber et al., 2000).

Although parasite studies on *Sardina pilchardus* have been conducted in different geographical regions, including the Mediterranean basin, the Northeast Atlantic and the Atlantic coastal waters of Northwest Africa (Paradižnik and Radujkovic, 2007; Rello et al., 2008; Marzoug et al., 2012; Shukhgalter, 2013; Shukhgalter and Lidvanov, 2018; Frigola-Tepe et al., 2024; Ramos et al., 2025) (Table 1) substantial knowledge gaps remain regarding regional parasite diversity, host–parasite relationships and spatial and seasonal variation in infection patterns in the Eastern Mediterranean and Black Sea regions.

**Table 1.** List of the parasite species detected in *Sardina pilchardus* in this study and their occurrences documented in different regions Table 1 long description.

Parasite	Region	References
**Digenea**		
*L. confusus*	Western Mediterranean	Shukhgalter, 1998
		Marzoug et al., 2012
	Northeast Atlantic	Shukhgalter and Lidvanov, 2018
		Shukhgalter, 2013
	Atlanto Iberian Waters	Ramos et al., 2025
	Black Sea	Present study
*A. stossichii*	Western Mediterranean	Reichenbach-Klinke, 1958
		Parukhin et al., 1971
		Orecchia and Paggi, 1978
		Marzoug et al., 2012
	Eastern Mediterranean	Papoutsoglou, 1976
	Adriatic Sea	Paradižnik, 1987
		Paradižnik and Radujkovic´, 2007
	Northeast Atlantic	Shukhgalter, 1998
		Shukhgalter, 2013
	Atlanto Iberian Waters	Ramos et al., 2025
	Black Sea	Present study
*P. ventricosa*	Western Mediterranean	Parukhin et al., 1971
		Parukhin, 1976
		Marzoug et al., 2012
	Atlanto Iberian Waters	Ramos et al., 2025
	Black Sea	Present study
*S. imparispine*	South Atlantic	Parukhin, 1975
*Stephanostomum* sp.	Mediterranean	Nikolaeva and Parukhin, 1969
*Stephanostomum* sp.	Black Sea	Present study
*Renicola* spp.	Mediterranean	Timon-David, 1953
*Renicola* sp.	Black Sea	Present study
**Nematoda**		
*H. aduncum*	Western Mediterranean	Reichenbach-Klinke, 1957
		Reichenbach-Klinke, 1958
		Orecchia and Paggi, 1978
		Rello et al., 2008
		Marzoug et al., 2012
	Eastern Mediterranean	Nikolaeva, 1964
	Mediterranean	Nikolaeva and Naidenova, 1964
		Santos et al., 2006
	Adriatic Sea	Sey, 1970
		Petter and Radujkovic, 1989
		Radujkovic and Raibaut, 1989
		Fioravanti et al., 1996
	Northeast Atlantic	Rello et al., 2008
	Northern Aegean Sea	Kuran et al., 2021
	Black Sea	Present study
**Coccidia**		
*E. sardina*	Northeast Atlantic	Pinto, 1956; Eiras, 2016
	Black Sea	Present study

In Türkiye, *Caligus* sp. has been reported from the Sea of Marmara, while *Hysterothylacium aduncum* and *Anisakis simplex* have been documented in this species in the Northern Aegean Sea (Demirhindi, 1961; Kuran et al., 2021) (Table 1). To date, however, no study has investigated the parasite diversity, abundance, host–parasite interactions or seasonal dynamics of *S. pilchardus* specifically in the Black Sea region and Türkiye.

In this context, the aims of this study are to (1) quantitatively determine the parasite species diversity and total abundance in *S. pilchardus*; (2) evaluate the relationship between parasite prevalence and abundance and the condition factor of the fish; (3) assess how parasite load varies according to sex, length groups and seasonal changes; and (4) to determine the level of co-occurrence in the parasite community by examining the pairwise relationships between parasite species.

## Materials and methods

### Fish sampling and analysis of parasites

In the present study, a total of 136 *Sardina pilchardus* individuals were obtained from commercial fishers along the Middle Black Sea coast of Türkiye (Samsun and Sinop provinces). Sampling was conducted monthly from October 2024 to May 2025. Due to the legal fishing ban during the summer months (June–August) and the unavailability of the species in September catches, the sampling period was restricted to 8 months. Consequently, seasonal data were categorized as follows: autumn (October and November), winter (December–February) and spring (March–May). The total length and body weight of the fish were measured in the laboratory, and their sex was recorded. Standard parasitological procedures were employed to examine both internal and external parasites. Initially, a comprehensive screening of the skin, fins, gills, mouth, liver, gallbladder, swim bladder, gonads, kidney, heart, mesenteries, oesophagus, stomach, pyloric caeca and intestine was conducted under a dissecting microscope. Subsequently, detailed examinations were performed using 2 types of preparations: scraping and squash. Scraped preparations were obtained from the skin, gills and gastrointestinal mucosa, while squashed preparations were prepared from internal organs (heart, liver, spleen, kidneys and gonadal tissue) to visualize potential pathogens in a translucent, thin film. All preparations were examined using a light microscope at 10×, 40× and 100× magnifications. The number of parasites was counted individually and the site of infection was recorded.

Parasite preparation procedures were carried out following the methods described by Paradižnik and Radujkovic´ (2007) for digeneans, Moravec (1994) for nematode and McGladdery (1987) for coccidia. All distinguishable parasites were counted and assigned to the most specific taxonomic category possible. Parasite species were identified following Gibson et al. (2002), Jones et al. (2005) and Bray et al. (2008) for digeneans; Moravec (1994) for nematode; and Lom and Dyková (1992) for coccidia.

Morphological studies were conducted and photographs were taken with the help of a trinocular light microscope (Olympus CX31, Tokyo, Japan) and equipped with a digital camera (Olympus BX53F, Tokyo, Japan) at Malatya Turgut Özal University, Faculty of Agriculture, Department of Aquaculture.

The total length of the examined fish ranged from 11 to 15.2 cm. *S. pilchardus* individuals were categorized into 5 length groups: L1 (11.0–11.9 cm), L2 (12.0–12.9 cm), L3 (13.0–13.9 cm), L4 (14.0–14.9 cm) and L5 (15.0–15.9 cm). The use of equal-width length classes in ecological studies reduces within-group variability and allows for a more precise detection of gradual changes in parasite abundance and diversity along the host size gradient (Timi et al., 2010). Therefore, narrow length intervals (1 cm) were used in this study to enable the identification of fine-scale patterns that might otherwise be obscured when using broader size groupings.

Fish condition was assessed using Fulton’s condition factor (*K*), calculated as *K =* 100 × *W L*^–3^, where *W* represents the body weight (g) and *L* represents the total length (cm). This index is based on the assumption of isometric growth. All measurements were obtained prior to the parasitological examination.

This study investigated the parasite community structure of *S. pilchardus* using an integrative ecological–parasitological framework.

### Parasitological indices and statistical analysis

The prevalence (P, %), mean intensity (MI) and mean abundance (MA) for parasites in sardine were determined according to Bush et al. (1997). The standard error (s.e.) for MI and MA was calculated. Prevalence is the percentage of infected fish among the total number of examined fish; MI is the average number of parasites per infected host; and MA is the average number of parasites per examined host, including both infected and uninfected fish. All statistical analyses were performed in Python (version 3.11). Data manipulation was conducted using pandas (version 2.x), while statistical analyses were carried out using *NumPy*. Graphical representations were generated using *Matplotlib*. Co-occurrence patterns among parasite species were assessed based on binary presence–absence data.

Total parasite abundance data were log10-transformed as log10(*x* + 1) prior to analysis. The association between total parasite infection and fish body condition was assessed using both Pearson’s *r* and Spearman’s *ρ* coefficients. For correlation analyses, 95% confidence intervals (CI) were calculated and reported to provide an estimate of the precision of the relationships.

Variations in parasite infracommunity composition were analysed using Bray–Curtis similarity on square-root transformed data, visualized through non-metric multidimensional scaling and statistically evaluated using Permutational Multivariate Analysis of Variance.

Parasite diversity was examined using observed species richness and the Shannon diversity index (*H*′), and sampling sufficiency was evaluated using species accumulation curves. Species accumulation curves were computed separately for each grouping factor (overall, length groups, seasons and sex) using randomized sample permutations (*N* = 136) to evaluate sampling sufficiency. The curves represent the mean cumulative parasite species richness as a function of the number of hosts examined, and variability around the mean is shown as ± 1 standard deviation (s.d.).

## Results

In this study, a total of 136 *Sardina pilchardus* were examined, and 7 parasite species were identified: 3 adult digeneans; *Pronoprymna ventricosa* Rudolphi, 1819, *Lecithaster confusus* Odhner, 1905, *Aphanurus stossichii* Monticelli, 1891, 2 metacercarial digeneans; *Stephanostomum* sp. and *Renicola* sp., 1 nematode; *Hysterothylacium aduncum* Rudolphi, 1802 (larvae) and 1 coccidian; *Eimeria sardinae* Thélohan, 1890 (sporulated oocysts). Regarding tissue tropism, the majority of the recovered parasites were localized in the visceral organs. Specifically, nematodes and trematodes were primarily found in the intestine (In), pyloric caeca (Pc), pharynx (Ph) and stomach (St), while *E. sardinae* oocysts were restricted to the testis (T), and *Stephanostomum* sp. metacercariae were found on the gills (G). Detailed microhabitat preferences for each species are presented in Table 2. The overall infection prevalence was 82.35%, the MI was 8.28 ± 0.74 and the MA was 6.82 ± 0.67. *Renicola* sp. was the most common (50%), *H. aduncum* the most abundant (3.43 ± 0.61) and *E. sardinae* the rarest species (0.73%). Sporulated oocysts of *E. sardinae* were observed only in the testicular squash preparations of a male fish (with a total length of 12.77 cm and weight of 14.88 g) caught in May 2025 (Table 2).

**Table 2.** Prevalence and infection metrics of parasites in *Sardina pilchardus* (*N* = 136) Table 2 long description.

Parasite species	Np	Nin	P (%)	MI ± s.e.	MA ± s.e	Min–Max	Site
**Digenea**							
*Lecithaster confusus*	10	5	3.68	2.00 ± 0.77^abc^	0.07 ± 0.04^γ^	1–5	Pc, In
*Aphanurus stossichii*	29	13	9.56	2.23 ± 0.41^ac^	0.21 ± 0.06^γ^	1–5	Ph, St
*Pronoprymna ventricosa*	167	34	25.00	4.91 ± 1.06^ab^	1.23 ± 0.31^β^	1–29	Pc, In
*Stephanostomum* sp.	19	15	11.03	1.27 ± 0.15^c^	0.14 ± 0.03^γ^	1–3	G
*Renicola* sp.	240	68	50.00	3.53 ± 0.38^a^	1.76 ± 0.24^α^	1–15	Pc, In
**Nematoda**							
*Hysterothylacium aduncum*	466	46	33.82	10.13 ± 1.36^b^	3.43 ± 0.61^αβ^	1–38	Pc, In, St
**Coccidia**							
*Eimeria sardinae*	–	1	0.73	–	–	–	T
**Total**	928	112	82.35	8.28 ± 0.74	6.82 ± 0.67	1–40	

Np, number of parasite individuals; Nin, number of infected fish; P (%), infection prevalence; MI, mean intensity; s.e.. standard error; MA, mean abundance; Min–Max, minimum–maximum parasite number; Pc, pyloric caeca; In, intestine; Ph, pharynx; St, stomach; G, gills; T, testis.

Within each column, values sharing at least one superscript symbol are not significantly different (P > 0.05), whereas values with no superscript symbols in common differ significantly (P < 0.05).

**Figure 1.**
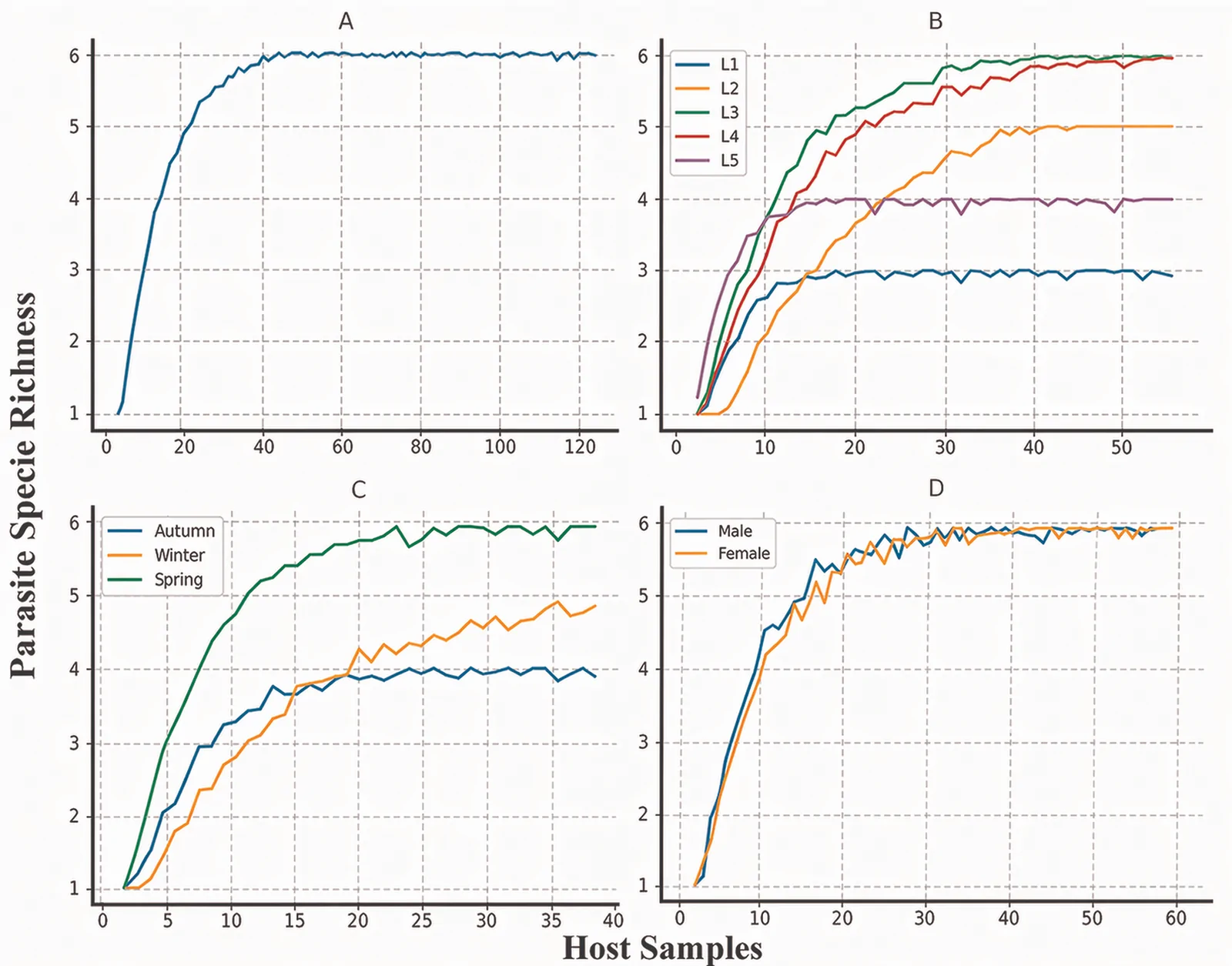
(A–D) Species accumulation curves showing parasite species richness in *Sardina pilchardus*: (A) total samples, (B) length groups and (C) seasons and (D) sexes. Curves were generated using randomized permutations (*N* = 136) and represent mean cumulative richness (±s.d.). Figure 1 long description.

*Eimeria sardinae* was excluded from the species accumulation analysis due to its extremely low prevalence and negligible abundance, as it was detected in only a single host; consequently, this species was not represented in the species accumulation curve. This suggests that the sampling effort was sufficient to capture the parasite community (Figure 1A).

Analyses of the *S. pilchardus* parasite community revealed significant variation primarily associated with host length and season (Figure 1B, 1C and Table 3).

**Table 3.** Parasite community metrics by sexes, length group and seasons in *Sardina pilchardus* Table 3 long description.

Metrics	Nf	Nin	Nps	Np	P (%)	MI ± s.e.	MA ± s.e.	*H*′
**Length groups (cm)**								
L1 (11.0–11.9)	13	8	3	107	61.54	13.37 ± 3.04^a^	8.23 ± 2.61^αβ^	0.88
L2 (12.0–12.9)	49	37	6	168	75.51	4.54 ± 0.55^b^	3.43 ± 0.50^β^	1.13
L3 (13.0–13.9)	43	37	6	291	86.05	7.86 ± 1.13^ab^	6.77 ± 1.06^αβ^	1.39
L4 (14.0–14.9)	31	30	6	362	96.77	12.06 ± 1.90^ac^	11.68 ± 1.88^α^	0.81
L5 (15.0–15.9)	5	5	4	38	100.00	7.60 ± 4.18^ab^	7.60 ± 4.18^αβ^	1.03
**Seasons**								
Autumn	29	20	4	86	68.96	4.30 ± 3.46^a^	2.96 ± 0.64^α^	1.20
Winter	50	36	5	264	72.00	7.33 ± 1.11^ac^	5.28 ± 0.92^α^	0.83
Spring	57	56	7	565	98.24	10.09 ± 1.22^bc^	9.91 ± 1.20^β^	0.79
Summer	*No fish specimens could be collected for parasitological examination during this season due to regulatory fishing closures in the sampling region*.							
**Sexes**								
Female	64	53	6	505	82.86	7.24 ± 0.83^a^	6.00 ± 1.14^α^	1.21
Male	70	58	7	420	82.81	9.53 ± 1.27^a^	7.89 ± 0.76^α^	1.24

Nf,number of examined fish; Nin, number of infected fish; Nps, number of parasite species; Np, number of parasite individuals; P (%), infection prevalence; MI, mean intensity; s.e., standard error, MA, mean abundance; H′: Shannon index.

Within each column, values sharing at least one superscript symbol are not significantly different (P > 0.05), whereas values with no superscript symbols in common differ significantly (P < 0.05).

Parasite infection parameters in *S. pilchardus* varied among length groups, with an overall increase in infection levels observed with increasing fish lenght. Total prevalence was lowest in the L1 group (61.54%) and highest in the L5 group (100%). *Renicola* sp. and *H. aduncum* were the most prevalent species across most length groups. In particular, *Renicola* sp. reached high prevalence values in larger fish (up to 80%), while *H. aduncum* showed its highest prevalence in the L4 group (67.74%). *Stephanostomum* sp., *Lecithaster confusus* and *Aphanurus stossichii* were not detected in the smallest length group. *P. ventricosa* was present in all length groups except the largest one, with its highest prevalence recorded in the smallest group (38.46%) (Table 4).

**Table 4.** Prevalence (P%), mean intensity (MI ± s.e.) and mean abundance (MA ± s.e.) of parasite species in *S. pilchardus* according to 5 length groups Table 4 long description.

		Fish length groups				
		L1	L2	L3	L4	L5
		(11.0–11.9 cm)	(12.0–12.9 cm)	(13.0–13.9 cm)	(14.0–14.9 cm)	(15.0–15.9 cm)
Parasite species	Infection parameters	(Nf = 13)	(Nf = 49)	(Nf = 43)	(Nf = 26)	(Nf = 5)
***L. confusus***	P (%)	0.00	0.00	9.30	3.22	0.00
	MI ± s.e.			2.25 ± 0.94	1.00 ± 0.00	
	MA ± s.e.			0.21 ± 0.13	0.03 ± 0.03	
***A. stossichii***	P (%)	0.00	4.08	16.28	12.90	40.00
			1.00 ± 0.00	2.57 ± 0.53	2.25 ± 0.95	1.00 ± 0.00
	MI ± s.e.		0.04 ± 0.02	0.42 ± 0.17	0.29 ± 0.17	0.40 ± 0.24
	MA ± s.e.					
***P. ventricosa***	P (%)	38.46	28.57	18.60	22.58	0.00
	MI ± s.e.	12.80 ± 5.10	2.50 ± 0.37	6.25 ± 1.91	2.57 ± 1.27	
	MA ± s.e.	4.92 ± 2.56	0.71 ± 0.19	1.16 ± 0.50	0.58 ± 0.33	
***Stephanostomum* sp.**	P (%)	0.00	8.16	9.30	22.58	20.00
	MI ± s.e.		1.25 ± 0.25	1.25 ± 0.25	1.28 ± 0.28	3.00 ± 0.00
	MA ± s.e.		0.11 ± 0.06	0.11 ± 0.06	0.29 ± 0.11	0.60 ± 0.60
***Renicola* sp.**	P (%)	30.77	51.02	46.51	61.29	80.00
	MI ± s.e.	8.50 ± 2.72	3.72 ± 0.63	3.30 ± 0.58	2.47 ± 0.50	2.75 ± 1.03
	MA ± s.e.	2.61 ± 1.36	1.90 ± 0.41	1.53 ± 0.37	1.52 ± 0.38	2.20 ± 0.97
***H. aduncum***	P (%)	23.08	16.32	32.55	67.74	40.00
	MI ± s.e.	3.00 ± 2.00	4.25 ± 0.96	10.21 ± 2.18	13.33 ± 2.31	11.00 ± 9.00
	MA ± s.e.	0.69 ± 0.53^α^	0.69 ± 0.27^α^	3.32 ± 1.01^α^	9.03 ± 1.92^β^	4.40 ± 3.92^αβ^
***E. sardinae***	P (%)	0.00	2.04	0.00	0.00	0.00
	MI ± s.e.		^a^			
	MA ± s.e.		^a^			
**Total**	P (%)	61.54	75.51	86.05	96.70	100.00
	MI ± s.e.	13.37 ± 3.04^a^	4.54 ± 0.55^b^	7.86 ± 1.13^ab^	12.06 ± 1.90^ac^	7.60 ± 4.18^ab^
	MA ± s.e.	8.23 ± 2.61^αβ^	3.43 ± 0.50^β^	6.77 ± 1.06^αβ^	11.68 ± 1.88^α^	7.60 ± 4.18^αβ^

Within each column, values sharing at least one superscript symbol are not significantly different (P > 0.05), whereas values with no superscript symbols in common differ significantly (P < 0.05).

a Sporulated oocysts were numerous in the preparations.

When infection parameters were evaluated across host length groups, infection prevalence was found to increase with host lenght. In contrast, metrics reflecting infection levels (MI and MA) differed significantly among length groups. Among the groups infected with 6 parasite species (L2, L3 and L4), both parameters reached their maximum values in L4 and their minimum in L2 (Table 3).

In contrast, the Shannon diversity index exhibited an inverse pattern (Table 3). Despite the relatively high species richness and infection levels observed in L4 (*H*′ = 0.81), this group showed the lowest Shannon index value, indicating an uneven distribution of parasite species and the dominance of particular taxa within the community. In this group, *H. aduncum* was clearly the dominant species. A similar pattern was observed in L1, where *P. ventricosa* was the predominant species (Table 4).

Conversely, the highest Shannon diversity index value was recorded in L3 (*H*′ = 1.39), where parasite species exhibited more comparable relative abundances, reflecting a more even community structure. Overall, these findings indicate that host size influences not only parasite species richness but also the evenness of species distribution within the parasite assemblage.

Water temperature varied seasonally during the sampling period (October 2024–May 2025), with mean monthly values ranging from 10 °C to 15 °C in spring, 15 °C to 25 °C in autumn and 8 °C to 15 °C in winter. Infection levels (98.24%), MA (9.91 ± 1.20) and MI (10.09 ± 1.22) were highest in spring, moderate in winter and lowest in autumn (Figure 1C). In *S. pilchardus*, statistically significant differences (*P* < 0.01) in parasite mean abundance were observed between spring and winter, as well as between spring and autumn (Table 3). When Shannon diversity was compared according to seasons, the lowest diversity was observed in spring (*H*′ = 0.79) despite having the highest species richness, Shannon diversity received the lowest value due to the strong dominance of *Renicola* sp. in this season and the highest diversity was seen in autumn (*H*′ = 1.20) and this indicates a more even distribution of parasite species (Table 3). Of all the digenean species, *Renicola* sp. had the broadest seasonal distribution, dominating in autumn and winter. Similarly, *P. ventricosa* increased during the colder seasons, with winter representing the peak period in terms of prevalence and intensity. While it occurred at moderate levels in autumn, its prevalence declined markedly in spring. *Stephanostomum* sp. was only observed in spring. *L. confusus* showed low prevalence overall and did not exhibit a clear seasonal trend. *A. stossichii* was not detected in autumn, showed very low prevalence in winter and increased noticeably in spring. The most prevalent species in spring was *H. aduncum* (Table 5).

**Table 5.** Prevalence (P%), mean intensity (MI ± s.e.) and mean abundance (MA ± s.e.) of parasite species in *Sardina pilchardus* according to seasons Table 5 long description.

	Seasons								
	Autumn			Winter			Spring		
	(Nf = 29)			(Nf = 50)			(Nf = 57)		
Parasite species	P (%)	MI ± s.e.	MA ± s.e.	P (%)	MI ± s.e.	MA ± s.e.	P (%)	MI ± s.e.	MA ± s.e.
*L. confusus*	10.34	2.33 ± 1.33	0.24 ± 0.18	2.00	1.00 ± 0.00	0.02 ± 0.02	3.51	1.50 ± 0.50	0.05 ± 0.04
*A. stossichii*	0.00	0.00	0.00	2.00	1.00 ± 0.00	0.02 ± 0.02**^β^**	19.30	2.33 ± 0.43	0.49 ± 0.15**^α^**
*P. ventricosa*	27.59	3.87 ± 1.27^ac^	1.07 ± 0.47	32.00	7.06 ± 2.01^bc^	2.66 ± 0.79	17.54	2.30 ± 0.71^a^	0.40 ± 0.17
*Stephanostomum* sp.	0.00	0.00	0.00	0.00	0.00	0.00	10.53	1.33 ± 0.33	0.14 ± 0.06
*Renicola* sp.	58.62	2.23 ± 0.48^a^	1.31 ± 0.35^αβ^	58.00	4.96 ± 0.70^b^	2.88 ± 0.53^β^	38.59	2.64 ± 0.46^a^	1.02 ± 0.24^α^
*H. aduncum*	10.34	4.00 ± 3.00^ab^	0.41 ± 0.34^α^	10.00	1.40 ± 0.40^a^	0.14 ± 0.07^α^	66.67	11.76 ± 1.50^b^	7.84 ± 1.24^β^
*E. sardinae*	–	–	–	–	–	–	1.75	*	*
**Total**	68.96	4.30 ± 3.46^a^	2.96 ± 0.64^α^	72.00	7.33 ± 1.11^ac^	5.28 ± 0.92^α^	98.24	10.09 ± 1.22^bc^	9.91 ± 1.21^β^

Within each column, values sharing at least one superscript symbol are not significantly different (P > 0.05), whereas values with no superscript symbols in common differ significantly (P < 0.05).

Although the MA and MI were slightly higher in males, no statistical difference was found between the sexes (*P* > 0.05). Parasite diversity was similar in both sexes (*H*′ = 1.21 and 1.24) (Tables 3, 6 and Figure 1D).

**Table 6.** Prevalence (P%), mean intensity (MI ± s.e.) and mean abundance (MA ± s.e.) of parasite species in *Sardina pilchardus* from Türkiye according to sex Table 6 long description.

	Sexes^a^					
	Female (Nf = 64)			Male (Nf = 70)		
Parasite species	P (%)	MI ± s.e.	MA ± s.e.	P (%)	MI ± s.e.	MA ± s.e.
*L. confusus*	3.12	3.00 ± 2.00	0.09 ± 0.07	4.28	1.33 ± 0.33	0.06 ± 0.03
*A. stossichii*	7.81	2.00 ± 0.77	0.16 ± 0.08	11.43	2.37 ± 0.50	0.27 ± 0.10
*P. ventricosa*	26.56	6.82 ± 1.93	1.81 ± 0.63	24.28	3.00 ± 0.69	0.73 ± 0.22
*Stephanostomum* sp.	10.94	1.29 ± 0.29	0.14 ± 0.05	11.43	1.25 ± 0.16	0.70 ± 0.05
*Renicola* sp.	53.12	3.21 ± 0.48	1.70 ± 0.32	47.14	3.87 ± 0.62	1.83 ± 0.37
*H. aduncum*	39.06	10.28 ± 2.04	4.01 ± 1.00	21.00	9.95 ± 1.78	2.99 ± 0.76
*E. sardinae*	–	–	–	1.43	–	–
**Total**	82.86	7.24 ± 0.83	6.00 ± 1.14	82.81	9.53 ± 1.27	7.89 ± 0.76

a Of the 136 examined fish, the sex of 2 individuals could not be determined and, therefore, they were excluded from the analyses.

**Figure 2.**
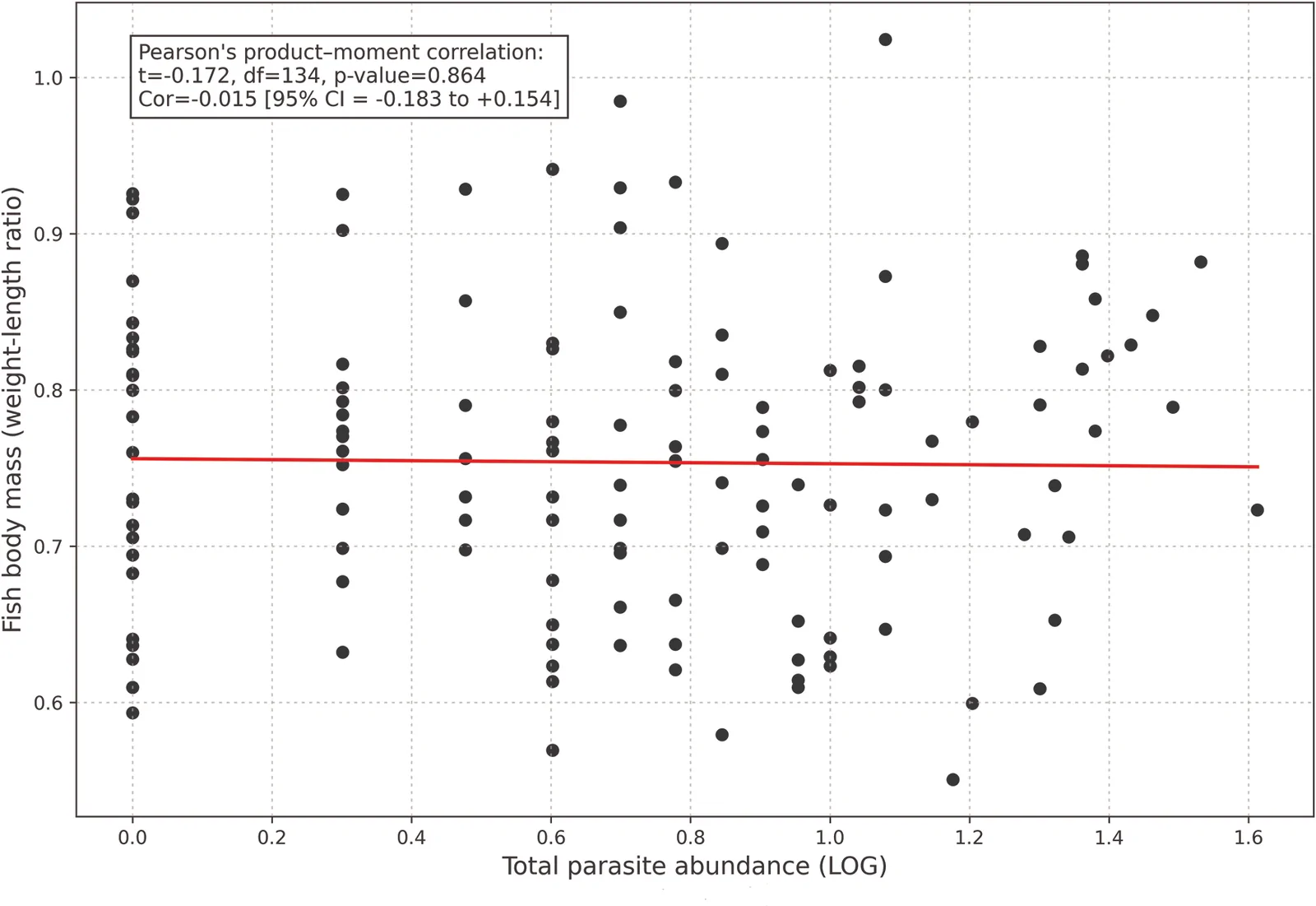
The relationship between total parasite abundance (log10-transformed) and fish body condition (Fulton’s *K*) in *Sardina pilchardus* was statistically non-significant (Pearson’s *r;P* > 0.05).

**Figure 3.**
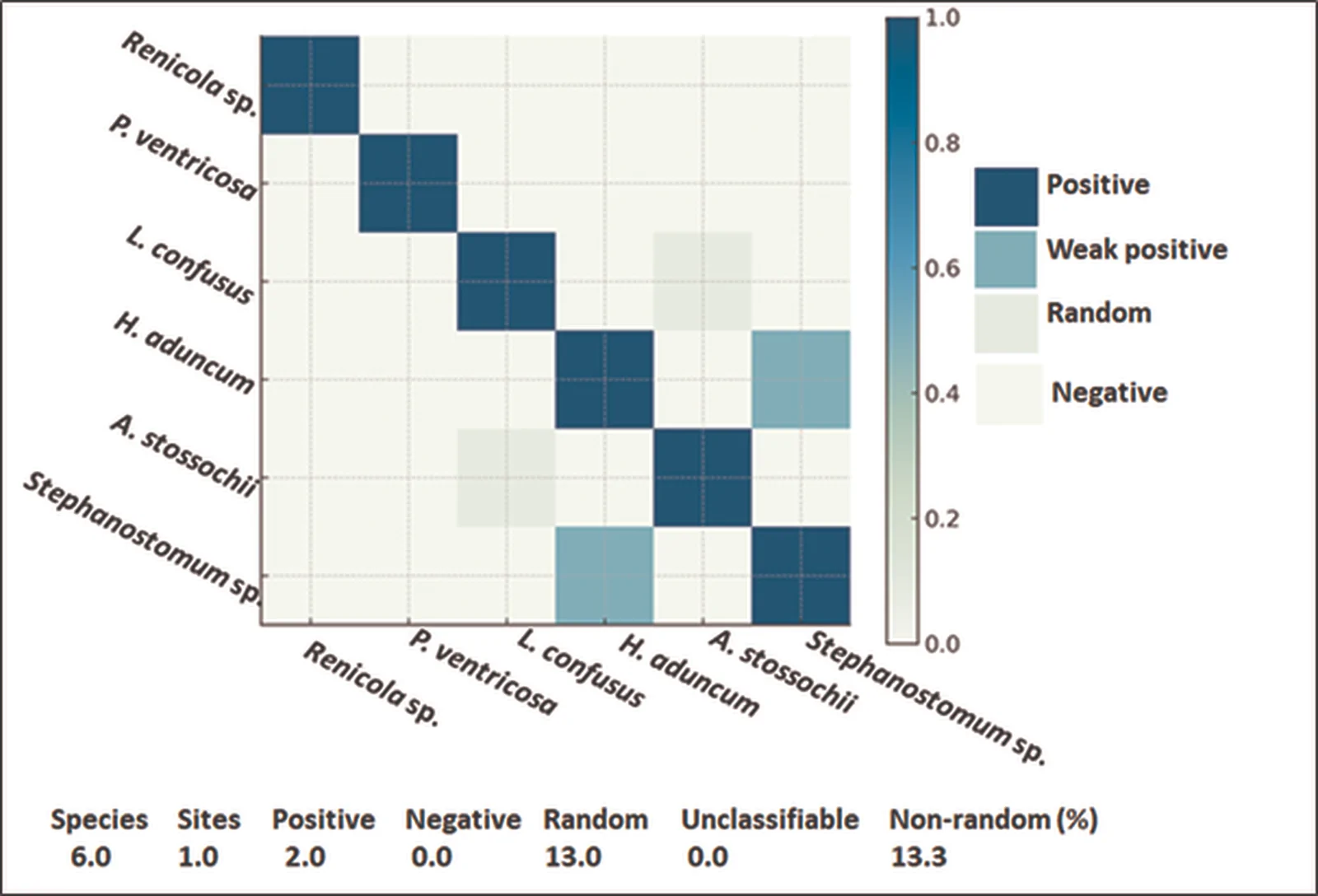
Co-occurrence matrix of parasite species detected in *Sardina pilchardus*, illustrating ecological associations among 6 parasite taxa. Each cell represents the interaction between a pair of species, catagorised positive (dark blue), weak positive (blue-green), random (light green) or negative (very light shading). Figure 3 long description.

There was no significant relationship between parasite abundance and body condition (Pearson’s *r* = − 0.015, *P* > 0.05) (Figure 2). Among the 15 pairwise comparisons, only 2 positively associated species pairs were identified: *H. aduncum* and *Stephanostomum* sp., which showed a moderate positive association (*r* = 0.494) and *L. confusus* and *A. stossichii*, which exhibited a weak positive association (*r* = 0.078). No negative associations were detected. Overall, the parasite community exhibited a weak co-occurrence pattern (Figure 3).

## Discussion

This study provides a detailed characterization of the parasite community of *Sardina pilchardus* along the Turkish Black Sea coast, emphasising the significance of parasite diversity, seasonal factors and host-specific characteristics in determining infection dynamics. The detection of 7 parasite species (5 digeneans, 1 nematode and 1 coccidian) indicates that the parasite fauna of sardines in this region is relatively rich.

When global and regional records are evaluated together, parasite diversity in *Sardina pilchardus* is known to vary markedly across regions. This largely reflects differences in environmental conditions, pelagic ecosystem dynamics and anthropogenic influences (Dessier et al., 2016; Ramos et al., 2025). Although approximately 39 parasite taxa have been reported across the species’ entire distribution range, this diversity is often considerably lower at the local scale (Dessier et al., 2016). Studies conducted in different areas within the same coastal system have further demonstrated that the parasite fauna of *S. pilchardus* exhibits pronounced spatial variability (Ramos et al., 2025). In addition, mesoscale ecosystem changes, particularly in coastal environments, may influence planktonic communities and, consequently, parasite assemblages through trophic transmission pathways (Shukhgalter and Lidvanov, 2018).

In the Atlantic and Atlanto-Iberian coastal waters, the reported parasite fauna is generally more diverse, with digenean trematodes, nematodes, myxosporans and coccidians being particularly prominent.

In western and southern Iberian waters, numerous taxa have been reported, including *Goussia* sp., *Kudoa* sp., *Aphanurus virgula, Aphanurus stossichii, Parahemiurus merus, Lecithaster confusus, Pronoprymna ventricosa, Pseudobacciger harengulae, Anisakis simplex* s.l., *Hysterothylacium* sp. and *Rhadinorhynchus* sp. (Ramos et al., 2025). Similarly, *Kudoa* sp. spores have been reported in the muscle tissue of *S. pilchardus* from northern Portugal (Cruz et al., 2011). In the Iberian sardine stock from the northern Spanish Atlantic coast, *Kudoa thyrsites* has also been recorded (Giulietti et al., 2024). In addition, ascaridoid nematodes such as *Anisakis simplex* s.s., *A. pegreffii* and *H. aduncum* have been reported from the Northeast Atlantic/Bay of Biscay and southern Portugal; Caballero-Huertas et al., 2023). These findings indicate that sardine populations of Atlantic origin may harbour a relatively high diversity of both helminth and protozoan/myxozoan parasites (Cruz et al., 2011; Dessier et al., 2016; Giulietti et al., 2024; Ramos et al., 2025).

In the Mediterranean basin, digenean trematodes and ascaridoid nematodes are particularly prominent. Along the western Mediterranean and Algerian coasts, digeneans including *Aphanurus stossichii, Aphanurus virgula, Lecithaster confusus* and *Pronoprymna ventricosa* have been reported (Marzoug et al., 2012). On the northwestern Mediterranean and Catalan coasts, *H. aduncum, Hysterothylacium* spp., *Contracaecum* spp. and, in some studies, *Anisakis* spp. have been recorded (Biton-Porsmoguer et al., 2020; Frigola-Tepe et al., 2022; Fuentes et al., 2022; Caballero-Huertas et al., 2023). The occurrence of species such as *Parahemiurus merus, Hysterothylacium aduncum* and *Anisakis pegreffii* in the Adriatic Sea indicates that the sardine parasite fauna in this region includes both digenean and nematode components (Zorica et al., 2016; Caballero-Huertas et al., 2023). However, in some Mediterranean studies, parasites were not identified to species level and were reported only as broader groups, such as trematode larvae or nematodes (Pennino et al., 2020). Therefore, methodological differences should be considered when making direct comparisons among Mediterranean subregions.

Records from the African coasts also vary according to region. Along the Mediterranean coast of North Africa, particularly in studies from Algeria and Tunisia, digeneans and copepod parasites are prominent. On the Algerian coast, digeneans such as *Aphanurus stossichii, A. virgula, Lecithaster confusus, Parahemiurus merus* and *Pronoprymna ventricosa* have been reported (Marzoug et al., 2012). On the Tunisian coast, *Peroderma cylindricum* has been reported as an important copepod parasite of sardine (Ktari and Abdelmouleh, 1980; Becheikh et al., 1994; Hajji et al., 2015). Along the Atlantic coast of Morocco, copepods such as *Peroderma cylindricum* and *Nothobomolochus cornutus* have been reported from sardine for the first time (Belghyti et al., 1997). This suggests that the parasite fauna of sardines along the African coasts may be shaped by both Mediterranean and Atlantic influences (Becheikh et al., 1994; Belghyti et al., 1997; Marzoug et al., 2012).

Regarding Türkiye and adjacent seas, previous studies have reported a more limited number of parasites in sardine. Records from Türkiye are mostly restricted to taxa such as *Hysterothylacium aduncum, Anisakis simplex* and *Caligus* sp. (Demirhindi, 1961; Kuran et al., 2021). Therefore, the detection of 7 parasite taxa in the present study, including 5 digeneans, 1 nematode and 1 coccidian, indicates that the parasite fauna of *S. pilchardus* in the Black Sea is richer than previously reported from Türkiye. Moreover, these findings suggest that the parasite community of Black Sea sardine shares some taxa with those reported from Mediterranean and Atlantic studies, while also exhibiting a regionally distinct composition.

In this study, with the exception of *E. sardinae*, the parasite species were characterized by complex life cycles involving trophic transmission through intermediate hosts. The detection of parasite species occurring in both adult and larval (e.g. metacercarial) stages in *S. pilchardus* indicates that this fish species serves as a definitive host for some parasites and as an intermediate host for others (Marcogliese, 2002). In this context, *S. pilchardus* can be considered not only as a recipient of infection but also as a vector facilitating the trophic transmission of parasites. This indicates that the structure and dynamics of parasite communities in pelagic systems are closely linked to host feeding ecology and trophic interactions.

Studies investigating parasite–host size relationships in clupeid fishes indicate that this relationship is neither unidirectional nor universal. While several studies have reported a positive relationship between fish size and parasite infection (Caballero-Huertas et al., 2023; Ramos et al., 2025), others have documented negative associations (Ferrer-Maza et al., 2016). In addition, the response of parasite communities to host size may vary among parasite taxa, with some species increasing and others decreasing in abundance as the host grows (Ogbon et al., 2023). Furthermore, it has been suggested that increases in infection intensity may reach a plateau beyond a certain host age or size. As in the present study, especially in studies where host age is not directly determined, larger fish may exhibit higher parasite loads not only due to age but also because of increased opportunities for exposure, dietary shifts or the provision of a larger and more suitable habitat for parasites (Habibi et al., 2018; Timi et al., 2020). Therefore, size–parasite relationships in clupeids should not be interpreted using a single linear model. Rather, these patterns should be considered context-dependent and shaped by multiple interacting factors, including trophic dynamics, environmental conditions and host biology (Poulin, 2000).

Parasite infection parameters in *Sardina pilchardus* exhibited clear variation in relation to host size. Overall, infection prevalence increased with increasing fish length, whereas MI and MA reached their highest levels in the intermediate length classes (particularly L4) and declined in the largest size group. This pattern suggests that although cumulative exposure time increases during host growth, infection dynamics may be shaped by a combination of factors, including ontogenetic dietary shifts, selective mortality and the development of more effective immune responses. Ontogenetic changes in feeding ecology appear to play a key role in structuring parasite transmission in sardines. Individuals in the smallest size class feed predominantly on small zooplankton, especially copepods and copepod eggs, which constitute the core of their diet. In intermediate size classes, zooplankton-based feeding persists but becomes more diverse, with increased consumption of decapod larvae and other larger planktonic organisms. In the largest individuals, a pronounced dietary shift has been reported, with copepods progressively replaced by larger zooplankton, particularly decapod larvae. Additionally, some studies have reported an increased contribution of phytoplankton, including diatoms and autotrophic dinoflagellates, to the diet of adult fish (Costalago and Palomera, 2014; Hure and Mustać, 2020).

The absence of *Stephanostomum* sp., *L. confusus* and *A. stossichii* in the smallest size group may indicate that the intermediate hosts involved in their life cycles are underrepresented in the diet of smaller individuals. Conversely, the absence of *L. confusus* and *P. ventricosa* in the largest size group may reflect not only biological processes, such as enhanced immune defenses with host growth (Šimková et al., 2008), but also sampling-related limitations, as rare yet heavily infected individuals may be overlooked in natural populations due to insufficient sampling effort (Poulin, 2000).

In contrast, the Shannon diversity index displayed a pattern distinct from species richness and infection levels. Despite relatively high species richness, the low diversity observed in the L4 group indicates dominance by a few parasite taxa and an uneven distribution within the community. In comparison, the L3 group exhibited a more balanced species composition, reflected in the highest diversity value. These findings emphasize that parasite communities should be evaluated not only in terms of species richness but also with respect to relative abundances and dominance structure.

In *S. pilchardus*, mean parasite abundance and MI differed significantly between spring and the other seasons (Table 3). In small pelagic fish such as *S. pilchardus*, parasite abundance and diversity do not respond directly to physicochemical fluctuations in the water column, but rather indirectly through trophic interactions and life cycle processes (Marcogliese, 2002, 2008). Among these factors, temperature plays a particularly important role by influencing both feeding activity and the life cycles of intermediate hosts. Seasonal variations in water temperature and nutrient availability in the Black Sea may therefore contribute to changes in parasite development rates and the population dynamics of intermediate hosts.

Although no specific studies on the feeding ecology of *S. pilchardus* have been conducted along the Black Sea coasts of Türkiye, studies on different clupeid fish species from this region have shown that feeding habits vary seasonally. These studies indicate that prey diversity and feeding activity increase during spring, as a wider range of planktonic groups being included in the diet, while certain prey groups become dominant in summer. In contrast, during autumn and winter, the diet is largely composed of a limited number of zooplankton groups, particularly copepods, and overall prey diversity decreases (Oven et al., 1997; Bayhan and Sever, 2015; Saglam and Yıldız, 2019; Bișinicu et al., 2024).

Studies conducted in different regions on *S. pilchardus* have also shown that feeding activity may vary seasonally, with changes being more pronounced during spring and summer; this pattern may be associated with increased primary production in spring, leading to greater availability of phytoplankton and small planktonic prey (Zorica et al., 2017; Bertrand et al., 2022).

In the present study, the higher infection intensity and parasite diversity observed in spring compared with autumn and winter may be associated with increased prey diversity and feeding activity during this period. Seasonal differences observed in infection parameters among parasite species may be explained by seasonal changes in the availability of intermediate hosts. Although increasing temperature has been reported to enhance cercarial release and infection rates, particularly in trematodes (Poulin, 2006), seasonal fluctuations in water temperature and nutrient availability may also lead to variations in parasite abundance (Marcogliese, 2008). Taken together, these findings suggest that season is an important factor influencing parasite transmission in sardines inhabiting the Black Sea.

Differences in parasitism between sexes may occur in fish due to behavioural, physiological, hormonal and diet-related factors (Poulin, 1996); however, in the present study, no significant differences were observed between male and female sardines in terms of parasite prevalence, MA or diversity (*P* > 0.05). The parasite species identified in the present study suggest that trophic transmission plays an important role, and it is well established that the diet of sardines largely depends on the environmental conditions of the region in which it occurs (Garrido et al., 2007, 2008; Costalago et al., 2015). Studies examining the relationship between sex and diet are limited; however, available evidence indicates that there are no significant differences between male and female sardines in terms of stomach fullness or dietary diversity (Garrido et al., 2008; Zorica et al., 2017). Considering the relationship between host size and parasitism in sardines, the nearly identical size distribution observed in the present sample, both in males (11–14.7 cm) and females (11.2–15.2 cm) suggests that individuals of both sexes are exposed to infective stages at comparable levels. Therefore, investigating the relationship between parasitism and sex in clupeid fishes through studies encompassing larger sample sizes and diverse ecosystems would help reduce existing uncertainties and enable more robust and reliable conclusions to be drawn.

No significant relationship was found between parasite abundance and body condition. The findings of the present study are consistent with previous studies showing that parasite infection has a weak and non-significant effect on condition factor in natural fish populations (Moreira et al., 2010; Hadjou et al., 2017; Biton-Porsmoguer et al., 2020; Hasegawa and Poulin, 2025). Many parasites that exert chronic and low virulence effects in natural populations may cause minimal or undetectable changes in body condition due to the physiological tolerance of the fish, and the inclusion of uninfected individuals in this calculation may mask adverse effects. The effect of parasites on condition factor can be more clearly understood through experimental studies (Hasegawa and Poulin, 2025).

The association between *H. aduncum* and *Stephanostomum* sp. was moderate, whereas the correlation between *L. confusus* and *A. stossichii* was weak, indicating a minimal tendency towards co-occurrence. No negative associations were observed. These results suggest minimal competitive interactions and that most species coexist independently. These findings are consistent with the weak coexistence, intermediate host effects and low niche overlap reported in parasite communities of marine fishes (Poulin, 2000). Co-occurrence analyses were performed on the pooled dataset without stratification by host size class, sex or season. This approach may obscure context-dependent associations, as co-occurrence patterns can vary with host ontogeny, environmental conditions and temporal dynamics. Future studies are recommended to examine size, sex and season-specific co-occurrence patterns in order to better elucidate the underlying ecological interactions.

In this study, the larval ascaridoids were considered most likely to belong to *Hysterothylacium aduncum* based on detailed morphometric criteria. This species has been widely and consistently reported as a common larva in numerous fish species throughout the Black Sea region based on morphological characterization. However, it is well established that larval stages of anisakid and raphidascaridid nematodes exhibit significant morphological overlap, which can complicate definitive species-level differentiation (Shamsi et al., 2013; Şimşek et al., 2018). While molecular characterization is essential to confirm these assignments with absolute certainty (Roca-Gerones et al, 2018), such genetic data could not be obtained in this study. Therefore, while our specimens are most likely *H. aduncum* based on local epidemiological data and regional literature, the lack of molecular validation remains a limitation and future studies incorporating genetic sequencing are warranted to fully clarify the species composition in this region.”

It should be noted that parasite detection in this study was based on visual examination. Although this approach is widely accepted for general parasitological surveys, its sensitivity may vary depending on the target parasite group, developmental stage, tissue location and the aims of the study. Complementary methods, such as enzymatic digestion or incubation, may be useful in some contexts for detecting small, live or embedded larval stages that are not easily observed by visual examination (McGladdery et al., 1986; Levsen et al., 2005; Shamsi and Suthar, 2016). Consequently, the parasite prevalence and intensity reported here may represent conservative estimates for some parasite taxa.

## Limitations of the study

Although this study provides significant insights into the parasite fauna of *S. pilchardus*, several limitations should be acknowledged. First, the sampling period was restricted to 8 months from October to May, due to legal fishing bans and seasonal unavailability, which precluded a full annual-cycle analysis. Second, the study was conducted along the central Black Sea coast (Samsun and Sinop provinces); therefore, the findings may not fully represent the entire Black Sea basin. Third, co-occurrence analyses were conducted on pooled data without stratification by lenght, sex or season, which may obscure context-dependent patterns. Finally, the use of visual examination for parasite detection may have provided a conservative estimate of the actual parasite load compared with more invasive methods, such as enzymatic digestion or incubation methods. Acknowledging these constraints ensures a transparent biological interpretation and provides a framework for future, more comprehensive investigations.

## Conclusion

In conclusion, the study identified 7 parasite species from *Sardina pilchardus* in the Black Sea coast of Türkiye, representing the first detailed research on this host’s parasite community in the region. The findings significantly expand current knowledge of the fish species’ parasite community and demonstrate that infection dynamics are influenced by host lenght and seasonal factors, while sex and body condition are less influential. These findings suggest that changes in plankton community structure, particularly those related to temperature regimes, may alter parasitism patterns and impact sardine population health. This may also have important implications for trophic transmission pathways and ecosystem functioning. Considering the shifting marine temperatures, long-term monitoring of this fish species is necessary. Such monitoring should incorporate integrated approaches including parasite surveys, feeding ecology and environmental parameters (e.g. temperature and plankton dynamics), and should be supported by seasonal sampling designs and studies focusing on intermediate host availability to better understand parasite transmission mechanisms.

For future research, comprehensive comparative approaches encompassing different salinity gradients and seasonal migration corridors are recommended to better elucidate the processes shaping host-specificity patterns and parasite recruitment. Such studies are critically important for detecting and monitoring of parasite species previously reported in *S. pilchardus* but not identified in the present study, and they will contribute to a more comprehensive understanding of host–parasite interactions in Black Sea sardines.

## Table 1 Long description

Parasite taxa detected in European sardine are listed by species, the regions where each has been reported, and supporting literature citations. Digenea includes L. confusus, A. stossichii, P. ventricosa, S. imparispine, Stephanostomum species, and Renicola species, reported across the Mediterranean, North East Atlantic, Atlanto Iberian waters, South Atlantic, and the Black Sea. The present study reports Black Sea occurrences for L. confusus, A. stossichii, P. ventricosa, Stephanostomum species, and Renicola species. Nematoda is represented by H. aduncum, which has the broadest geographic coverage, appearing in multiple Mediterranean subregions, the North East Atlantic, the Northern Aegean Sea, and also the Black Sea in the present study. Coccidia includes E. sardina, previously reported from the North East Atlantic and recorded in the Black Sea by the present study. Regional labels vary in specificity, so comparisons should be interpreted as literature presence records rather than standardized prevalence or intensity measures.

Navigate back to Table 1.

## Table 2 Long description

Parasite infection metrics are reported for 136 Sardina pilchardus, listing parasite counts, number of infected fish, prevalence, mean intensity and abundance with standard errors, ranges, and infection sites. Overall, 112 fish were infected (82.35 percent), with 928 parasite individuals recorded and a total range of 1 to 40 parasites per fish. Renicola sp. had the highest prevalence at 50 percent (68 fish infected) and 240 individuals, occurring in pyloric caeca and intestine. Hysterothylacium aduncum showed the highest mean intensity at about 10 parasites per infected fish, with 466 individuals, 33.82 percent prevalence, and sites in pyloric caeca, intestine, and stomach. Pronoprymna ventricosa was also common (25 percent prevalence; 167 individuals) in pyloric caeca and intestine, while Aphanurus stossichii and Stephanostomum sp. had lower prevalence (about 10 to 11 percent) in pharynx and stomach, and gills, respectively. Lecithaster confusus was rare (3.68 percent prevalence), and Eimeria sardinae was detected in one fish only, with parasite counts and intensity not reported. Superscript letters next to some intensity and abundance values indicate groups that are not meaningfully different from each other, so small differences among those marked values should not be over-interpreted.

Navigate back to Table 2.

## Table 3 Long description

Parasite community metrics are summarized for sardines by length group, season, and sex, including numbers examined and infected, parasite species and individuals, prevalence, mean intensity and abundance with standard errors, and Shannon diversity. By length, prevalence rises from 61.54% in L1 to 96.77% in L4 and 100% in L5; parasite individuals peak in L4 at 362, and mean abundance is highest in L4 at 11.68 with a standard error of 1.88. Mean intensity is highest in L1 at 13.37 with a standard error of 3.04 and is also high in L4 at 12.06 with a standard error of 1.90, while L2 has the lowest intensity at 4.54 with a standard error of 0.55. Shannon diversity is highest in L3 at 1.39 and lowest in L4 at 0.81. By season, spring has the highest prevalence at 98.24% and the most parasite individuals at 565, with mean abundance 9.91 and a standard error of 1.20; autumn shows the lowest prevalence at 68.96 and the fewest parasite individuals at 86. No summer data are available because fish could not be collected during seasonal fishing closures. By sex, females and males have nearly identical prevalence, about 82.8%, but males show higher mean intensity and abundance (9.53 and 7.89) than females (7.24 and 6.00), with similar Shannon diversity around 1.2. Superscript letters indicate which values are not meaningfully different within the same metric, and small sample size in L5 may limit comparisons.

Navigate back to Table 3.

## Table 4 Long description

Parasite infection metrics are reported for sardines grouped by length from 11.0 to 15.9 cm, with sample sizes of 13, 49, 43, 26, and 5 fish. For each parasite species, the table lists prevalence percent plus mean intensity and mean abundance with standard error by length group. Total prevalence increases steadily with size, from 61.54 percent in the smallest fish to 100 percent in the largest; total mean abundance is highest in the 14.0 to 14.9 cm group at 11.68 with standard error 1.88. Renicola sp. is the most consistently prevalent parasite, rising from 30.77 percent in the smallest group to 80.00 percent in the largest, with mean intensity generally around 2.47 to 8.50. H. aduncum shows a strong size-related increase, peaking at 67.74 percent prevalence and mean abundance 9.03 with standard error 1.92 in the 14.0 to 14.9 cm group, then dropping to 40.00 percent prevalence in the largest group. P. ventricosa is common in smaller fish at 38.46 percent prevalence but declines to zero in the largest group, while Stephanostomum sp. increases to about 22.58 percent in the 14.0 to 14.9 cm group and remains 20.00 percent in the largest. L. confusus is rare, appearing only in the middle groups, and E. sardinae is recorded only in the 12.0 to 12.9 cm group at 2.04 percent with other values not provided. Superscript letters indicate which mean values are not statistically different, and the largest length group has a very small sample size, so its estimates are less stable.

Navigate back to Table 4.

## Table 5 Long description

Seasonal parasite metrics in Sardina pilchardus are reported for autumn (29 fish), winter (50), and spring (57), including prevalence percent, mean intensity with standard error, and mean abundance with standard error for each parasite species plus totals. Overall infection is highest in spring, with total prevalence 98.24 percent, mean intensity 10.09, and mean abundance 9.91; autumn and winter totals are lower at 68.96 and 72.00 percent prevalence, with mean abundance 2.96 and 5.28. H. aduncum drives the spring peak, rising to 66.67 percent prevalence with mean intensity 11.76 and mean abundance 7.84, compared with about 10 percent prevalence and much lower abundance in autumn and winter. Renicola sp. is common in all seasons, with prevalence near 59 percent in autumn and winter and 38.59 percent in spring; winter shows the highest Renicola mean intensity and abundance (4.96 and 2.88). P. ventricosa occurs in all seasons, peaking in winter for prevalence (32.00 percent) and mean abundance (2.66), and dropping in spring (17.54 percent prevalence; 0.40 mean abundance). A. stossichii is absent in autumn, low in winter (2.00 percent), and higher in spring (19.30 percent), while Stephanostomum sp. appears only in spring (10.53 percent). E. sardinae is recorded only in spring at 1.75 percent, with intensity and abundance not provided. Superscript letters indicate which values are not statistically different, so comparisons should consider those groupings rather than small numeric differences alone.

Navigate back to Table 5.

## Table 6 Long description

Sex-specific parasite metrics are reported for Sardina pilchardus from Türkiye, giving prevalence (percent infected), mean intensity with standard error, and mean abundance with standard error for females (64) and males (70). Overall prevalence was nearly identical between sexes, about eighty three percent in both, but males had higher total mean intensity (about 9.5) and mean abundance (about 7.9) than females (about 7.2 intensity and 6.0 abundance). Renicola sp. was the most prevalent parasite in both sexes, affecting about fifty three percent of females and forty seven percent of males, with similar mean abundance around 1.7 to 1.8. H. aduncum was also common, higher in females (about thirty nine percent) than males (about twenty one percent), and had the highest mean intensity in both sexes (about ten). P. ventricosa showed moderate prevalence in both sexes (about twenty five to twenty seven percent) but higher intensity and abundance in females than males. L. confusus and A. stossichii were low-prevalence parasites in both sexes, and E. sardinae appeared only in males at very low prevalence with no intensity or abundance values reported. Two fish with undetermined sex were not included, so results apply only to sexed individuals.

Navigate back to Table 6.

## Figure 1 Long description

The image A showing a line graph with the vertical axis labeled Parasite Species Richness and tick labels 1, 2, 3, 4, 5, 6. The horizontal axis shows tick labels 0, 20, 40, 60, 80, 100, 120. One line is plotted. Readable coordinate pairs: (0, 1), (20, 5), (40, 6), (60, 6), (80, 6), (100, 6), (120, 6). The line rises from 1 at 0 to 5 at 20 and reaches 6 at 40, then stays at 6 through 120. The image B showing a line graph with the vertical axis tick labels 1, 2, 3, 4, 5, 6. The horizontal axis shows tick labels 0, 10, 20, 30, 40, 50. A legend lists L1, L2, L3, L4, L5. Five lines are plotted. L1: (0, 1), (10, 3), (20, 3), (30, 3), (40, 3), (50, 3). L2: (0, 1), (10, 2), (20, 3), (30, 4), (40, 5), (50, 5). L3: (0, 1), (10, 4), (20, 5), (30, 5), (40, 6), (50, 6). L4: (0, 1), (10, 4), (20, 5), (30, 6), (40, 6), (50, 6). L5: (0, 1), (10, 4), (20, 4), (30, 4), (40, 4), (50, 4). L4 reaches 6 by 30 and stays at 6. L3 reaches 6 by 40 and stays at 6. L2 reaches 5 by 40 and stays at 5 through 50. L5 reaches 4 by 10 and stays at 4 through 50. L1 reaches 3 by 10 and stays at 3 through 50. The image C showing a line graph with the vertical axis labeled Parasite Species Richness and tick labels 1, 2, 3, 4, 5, 6. The horizontal axis shows tick labels 0, 5, 10, 15, 20, 25, 30, 35. A legend lists Autumn, Winter, Spring. Three lines are plotted. Autumn: (0, 1), (5, 3), (10, 4), (15, 4), (20, 4), (25, 4), (30, 4), (35, 4). Winter: (0, 1), (5, 2), (10, 3), (15, 4), (20, 4), (25, 5), (30, 5), (35, 5). Spring: (0, 1), (5, 4), (10, 5), (15, 6), (20, 6), (25, 6), (30, 6), (35, 6). Spring reaches 6 by 15 and stays at 6 through 35. Winter reaches 5 by 25 and stays at 5 through 35. Autumn reaches 4 by 10 and stays at 4 through 35. The image D showing a line graph with the vertical axis tick labels 1, 2, 3, 4, 5, 6. The horizontal axis is labeled Host Samples and shows tick labels 0, 10, 20, 30, 40, 50, 60. A legend lists Male and Female. Two lines are plotted. Male: (0, 1), (10, 5), (20, 6), (30, 6), (40, 6), (50, 6), (60, 6). Female: (0, 1), (10, 5), (20, 6), (30, 6), (40, 6), (50, 6), (60, 6). Both lines rise from 1 at 0 to 5 at 10, reach 6 at 20 and stay at 6 through 60.

Navigate back to Figure 1.

## Figure 3 Long description

Square heatmap matrix with the same six labels on both axes: Renicola sp., P. ventricosa, L. confusus, H. aduncum, A. stossichii, Stephanostomum sp. A legend at right lists four categories: Positive, Weak positive, Random, Negative. Cells on the main diagonal form a continuous line of filled squares from the top-left to the bottom-right. Off-diagonal filled cells appear in the lower-right portion of the matrix. One filled cell is at the intersection of the row H. aduncum and the column Stephanostomum sp. Another filled cell is at the intersection of the row A. stossichii and the column L. confusus. Additional filled cells appear around the intersections involving A. stossichii and Stephanostomum sp. A vertical scale bar beside the legend is labeled from 0.0 at the bottom to 1.0 at the top. A table below the heatmap shows column headers: Species, Sites, Positive, Negative, Random, Unclassifiable, Non-random (percent). The first visible row shows: 6, 15, 2, 0, 13, 0, 13.0.

Navigate back to Figure 3.
